# Association between quality of life, sleep quality and mental disorders in Iranian older adults

**DOI:** 10.1038/s41598-022-20013-0

**Published:** 2022-09-20

**Authors:** Ali Khorshidi, Marzieh Rostamkhani, Roya Farokhi, Abbas Abbasi-Ghahramanloo

**Affiliations:** 1 Department of Epidemiology, School of Health, Ilam University of Medical Sciences, Ilam, Iran; 2 Department of Psychology, School of Medicine, Ilam University of Medical Sciences, Ilam, Iran; 3 Department of Public Health, School of Health, Ardabil University of Medical Sciences, Ardabil, Iran

**Keywords:** Diseases, Risk factors, Health care, Public health, Quality of life

## Abstract

Aging as a major challenge can affect the development and growth of countries all around the world. This study aimed to identify the subgroups of the elderly based on the quality of life (Qol), sleep quality, and common mental disorders and assess the role of demographic characteristics on the membership of participants in each latent class. This cross-sectional study was conducted on 1064 people over the age of 60 years. The sample was selected through cluster sampling in northern Iran. All participants completed six sets of checklists and questionnaires. Data analysis was performed using latent class analysis. Three latent classes were identified; namely, (1) healthy (66.8%), (2) anxious and with poor sleep quality (28.6%), and unhealthy (4.6%). Being Female significantly increased the odds of membership in classes 2 and 3 compared to class 1. Furthermore, living in urban areas increased the odds of belonging to class 2 and class 3 compared to class 1. Illiteracy was also shown to increase the odds of being in class 3 in comparison to class 1. Results from the present indicate that the co-occurrence of health problems in 33.2% of the elderly was observed in various forms. The results of this study can be used in prioritizing health programs for the elderly and emphasizing high-risk groups.

## Introduction

Aging as a major challenge can affect the development and growth of countries all around the world, and causes social and economic transformation^1^. Currently, much attention and effort have been paid to find effective strategies aimed to help the elderly to achieve successful aging^2^. Researchers have estimated that according to the increasing trend of population growth over the next years, approximately 16% of the world's population will be 65 years and older by 2050^3^. In Iran, the elderly population was reported as ten million by 2019^4^. It is predicted that a quarter of the total Iranian population will be over age 60 years by 2050^5^.

Elderly people normally face many health problems, among which, poor sleep quality and mental disorders, such as anxiety and depression are more prevalent, which negatively affect the quality of life (QoL) of these individuals gradually^6,7^. In recent years, due to the increase in the elderly population, the QoL and performance of these people have become significantly important and drawn much attention^8^. Accordingly, low QOL is associated with reduced social functioning and the inability to provide basic needs among the elderly^9^. Of course, it should be mentioned that QoL is a latent variable that cannot be directly measured and an appropriate method should be used that can evaluate one's QoL in all dimensions^6^.

Another common problem in the elderly is poor sleep quality. This can increase the level of fatigue and reduce the QoL and increase cardiovascular disease (CVD) and psychiatric disorders^7^. Due to the occurrence of numerous health problems in the elderly, sleep disorders are also exacerbated in these people, which explain more frequency of sleep problems amongst the elderly^10^.

Another important aging-related health problem is mental health. These people are more likely to develop mental disorders due to the higher probability of occurrence of different types of problems among them^11^. The lifetime and current prevalence of mental disorders in the elderly has been reported as ranging from 1 to 18%^12,13^. Depression is also a common condition in older ages, which may affect the prognosis of other chronic diseases and increase disability^14^. Psychiatrists believe that in the elderly, combined occurrence of depression and anxiety is common, that is depressive symptoms are displayed by 90% of patients with anxiety, and the co-occurrence of these two medical conditions may appeal in more severe and persistent forms^15^. The prevalence of depression has been reported as 34.6–77.5% in the elderly^16^. ADHD has severe negative impact on daily life functioning such as impairments in academic, social functioning and occupational, in addition ADHD has also been associated with lower quality of life^17^. Previous studies have found that ADHD is associated with psychiatric comorbidities, which complicate the clinical manifestation of the disorder^18,19^. Individuals with ADHD may experience increased difficulties as the demands of life increase, which may contribute to the development of anxiety and depression^20^. For assessment of the health problems in the elderly, integrated models are needed to provide an overview of the status of these problems^6^. As such, Latent Class Analysis (LCA) is an effective technique to identify health-related problems and examine their subgroups among older adults. This approach can be used to effectively assess the structure of heterogeneous diagnostic institutions and identify their subgroups in a systematic way^10^. This approach has been used to identify different clusters of sleep problems^10,21^ and subgroups of psychiatric disorders in the elderly^22^. In the present study, using LCA, the elderly subgroups were identified based on QoL, sleep quality, and common mental disorders, and finally, their co-occurrence pattern was measured. Also, the effect of demographic variables on the odds of latent class membership was examined.

## Methods

This population-based study recruited a total of 1093 people over the age of 60 years by using cluster sampling in northern Iran. Samples were selected from the population affiliated with urban and rural health centers. Data were collected using questionnaires and interviews in health centers. If the person was not able to go to the health center, the interviewers would go to the person's home to gather the required information. Prior to the interview, the interviewees provided the elderly with the necessary explanations about the objectives of the study and were assured that the questionnaires would be anonymous, and the study participation is totally voluntary.

Six checklists and questionnaires were completed for all participants. In the first part, demographic information, such as age, gender, etc. were evaluated. The second part assessed Attention Deficit Hyperactivity Disorder (ADHD), which was measured using the Persian version of the 30-item Conner's Adult ADHD Rating Scales. According to this scale, subscale D was considered as the total score of ADHD, the overall score of this subscale varies from 0 to 36, and a score above 15 was considered as “yes”^23,24^.

The third part of our questionnaire assessed depression, anxiety, and stress, by using the DASS-21 questionnaire. This questionnaire consisted of 21 items (7 questions per scale). The questions were scored 0–3, and for depression, anxiety, and stress, respectively scores of 10 or higher, 8 or higher, and 15 or higher were considered as having these disorders^25^. This questionnaire was validated for Iran’s population^26^.

Additionally, the 12-item SF-12 was used to measure the status of QoL of the elderly. The QoL SF-12 is a shortened form of the 36-item QOL questionnaire. The scores of QoL SF-12 ranged from 12 to 48, which higher scores indicating better QoL. Scores of 12–24 were considered as poor QoL, 25–36 as moderate QoL, and scores of 37–48 were considered as good QoL^27^. This questionnaire was validated for adults in Iran^28^.

The fifth part of the questionnaire assessed sleep quality among the elderly. This variable was measured using the Pittsburgh Sleep Quality Index (PSQI), a standard 18-item scale. Accordingly, a score of 0–4 indicated good sleep quality, and 5 and higher represented poor sleep quality^29^. The validity and reliability of the questionnaire were confirmed in Iran^30^.

Finally, the last part of the questionnaire was related to obsessive–compulsive disorder (OCD) assessment which was measured by the revised OCD scale (OCI-R). This scale with 18 items, measures the symptoms of OCD. The minimum score is 0 and the maximum is 72. A score between 0 and 24 indicated low OCD, between 24 and 36 as moderate OCD, and a score above 36 indicated high OCD^31^. In Iranian studies, the validity and reliability of this questionnaire were confirmed^32^.

### Statistical analysis

Using the LCA, the latent classes of the latent variable were identified based on some indicator variables. To select the best model, the G2 index was first calculated, which was similar to the Chi-square index. Also, Akaike information criteria (AIC) and Bayesian Information Criterion (BIC) were calculated. For all of these measures, lower scores reflected better model fitting and parsimony. Item response probabilities higher than 0.5 were used to label the latent classes and describe the characteristics of each class. Seven observed variables were selected for subgrouping the elderly. These variables were ADHD, depression, anxiety, stress, OCD, sleep quality, and QoL. After the model was finalized, we entered covariates in the LCA model. These variables were age, gender, marital status, area of living, and education level. As shown in Table 1, the indices of final model selection for each of the LCA models were calculated. As such, the three-class model had the lowest AIC and BIC among all models. Considering these criteria as well as the ability to interpret the results, finally, the three-class model was preferred for subgrouping the elderly.

**Table 1:** Comparison of LCA models with different latent classes based on model selection statistics.

Number of latent class	Number of parameters estimated	G^2^	df	AIC	BIC	Entropy	Maximum log-likelihood
1	7	759.93	120	773.93	808.72	1.00	− 2510.58
2	15	148.62	112	178.62	253.17	0.89	− 2204.92
**3**	**23**	**51.63**	**104**	**97.63**	**211.93**	**0.70**	− **2156.43**
4	31	36.62	96	98.62	252.68	0.68	− 2148.92
5	39	27.78	88	105.78	299.60	0.69	− 2144.51
6	47	23.18	80	117.18	350.76	0.73	− 2142.20
7	55	17.93	72	127.93	401.27	0.72	− 2139.58

The final model is in bold.

LCA, latent class analysis; AIC, Akaike information criterion; BIC, Bayesian information criterion.

SPSS version 16.0 was used for simple statistical analysis. LCA was performed using SAS version 9.4. In all analyses, the P-value < 0.05 was considered statistically significant.

### Ethics approval and consent to participate

The study was approved by the Ethics Committee of Ilam University of Medical Sciences (IR.MEDILAM.REC.1399.276). Permission on conduct the study was obtained from this committee and all staff had signed an informed consent from. All literate participants had signed an informed consent form and also for illiterate participants legal guardians completed and singed informed consent form. In this study all procedures were performed in accordance with declaration of Helsinki.

## Results

Out of 1093 invited individuals, a total of 1064 were participated in the study, bringing the response rate of 97%. The average age of the participants was 68.7 ± 7.5 years. Also, more than 51% of the subjects were males, 39.4% were illiterate, the majority of them were married (94.9%), and 51.1% were living in urban areas. Table 2 represented the demographic characteristics of the study participants. Table 3 summarized the most common health problems in older adults. Besides, 50.4% had poor sleep quality and 37.8% had anxiety. Moreover, some problems were less prevalent. For example, among the elderly, only 1.6% had severe OCD.

**Table 2:** Demographic characteristics of Iranian older adults.

Items	Total (1064) N (%)
**Age group (years old)**	
60–75	886 (83.3)
76–90	170 (16.0)
> 90	8 (0.8)
**Gender**	
Female	520 (48.9)
Male	544 (51.1)
**Marital status**	
Single	7 (0.7)^a^
Married	1008 (94.9)
Others	47 (4.4)
**Living place**	
Rural	48.9 (520)
Urban	51.1 (544)
**Education**	
Illiterate	419 (39.4)^a^
Elementary and higher	644 (60.6)

^a^There were missing values. We calculated the prevalence on valid data.

**Table 3:** Quality of life, sleep quality and common mental disorders among Iranian older adults.

Items	Total (1064) n(%)
**ADHD**	
No	1041 (98)^a^
Yes	21 (2.0)
**Depression**	
No	961 (90.3)
Yes	103 (9.7)
**Anxiety**	
No	662 (62.2)
Yes	402 (37.8)
**Stress**	
No	1015 (95.4)
Yes	49 (4.6)
**OCD**	
Low	968 (91.0)
Moderate	79 (7.4)
Severe	17 (1.6)
**Quality of life**	
Poor	22 (2.1)
Moderate	336 (31.6)
Good	706 (66.4)
**Sleep quality**	
Poor	535 (50.4)^a^
Good	526 (49.6)

^a^There were missing values. We calculated the prevalence on valid data.

Table 4 shows the results of the three-class LCA model. Based on these findings, older adults were divided into three groups. The first class (healthy) comprised 66.8% of the participants. Also the second class (anxious and with poor sleep quality) and third class (unhealthy) described 28.6% and 4.6% of the study participants respectively. In latent class 1 (unhealthy) the probability of occurrence of all examined items was low. Besides, in the third latent class (unhealthy), all health problems except ADHD and low QoL were most likely to occur. Another class demonstrated a different pattern of common problems among older adults. Those with second latent class membership were more likely to have anxiety and poor sleep quality.

**Table 4:** Three latent class model of quality of life, sleep quality and common mental disorders among Iranian older adults.

Items	Latent class		
	Healthy	Anxious and poor sleep quality	Unhealthy
Latent class prevalence	0.668	0.286	0.046
**Item-response probabilities**			
Having ADHD	0.000	0.006	0.390
Having depression	0.003	0.180	**0.943**
Having anxiety	0.171	**0.764**	**0.972**
Having stress	0.000	0.034	**0.791**
Having OCD	0.016	0.172	**0.655**
Having poor sleep quality	0.340	**0.816**	**0.954**
Having weak quality of life	0.004	0.014	0.306

Item-response probabilities > .5 in bold to facilitate interpretation.

Table 5 shows the odds ratio of membership in each class compared to first-class, associated with demographic characteristics. This index compares the odds of membership in each class with the reference class (e.g. first class). As shown in Table 5, the female gender significantly increased the odds of membership in classes 2 and 3 compared to class 1. Furthermore, living in urban areas increased the odds of belonging to class 2 and class 3 compared to class 1. Illiteracy was also shown to increase the odds of being in class 3 in comparison to class 1.

**Table 5:** Predictors of membership in latent classes of quality of life, sleep quality and common mental disorders among Iranian older adults.

Predictors	P-value	Healthy	Anxious and poor sleep quality	Unhealthy
			OR (95%CI)	OR (95%CI)
Age (above 75 years v.s.75 and under 75)	0.7697	Reference	1.22 (0.73–2.03)	1.16 (0.55–2.47)
Sex (female v.s. male)	< 0.001	Reference	2.83 (1.89–4.23)	2.29 (1.24–4.23)
Marital status (single v.s. married/others)	0.4927	Reference	2.16 (0.46–10.07)	0.17 (0.0–498.94)
Place of living (urban citizen)	< 0.001	Reference	2.03 (1.34–3.05)	4.72 (2.40–9.27)
Education (illiterate v.s. elementary and higher)	0.0254	Reference	1.17 (0.77–1.79)	2.47 (1.33–4.57)

## Discussion

The results of this study showed the status of quality of life, sleep quality, and the prevalence of each of the mental disorders among older adults. In this study, three distinct classes were identified for the elderly, "healthy" as class 1, "anxious with poor sleep quality" as class 2, and "unhealthy" as class 3. Each of these classes had specific characteristics. In the second class, for example, the probability of having anxiety and low QoL was high, while in the third class the probability of having depression, anxiety, stress, OCD, and poor sleep quality was high. Therefore, it can be concluded that 4.6% of the subjects had a wide range of aging problems simultaneously. However, 28.6% of the participants were members of anxiety with low quality of life class. The LCA approach in the elderly was used to investigate some of these problems. Although no previous similar study was found, several studies on the older adults using this method are presented here:

Chen et al. used the LCA to examine the subgroups of poor sleep quality in the older adults in Taiwan. In this study, 3 classes, high insomnia, mild insomnia, and high hypnotics were identified for low sleep quality^33^. In another study, Curran et al.^15^ used this approach to identify the profile of anxiety and depression in the Irish elderly. In this study, four classes were identified: low, comorbidity, anxiety, and subthreshold depression. Due to the differences between these studies and the present study, the results are not comparable. However, it is noteworthy that in terms of sleep quality and mental disorders, there were separate classes for the elderly, some of which suffered from severe complications of these disorders. In the present study, older adults of latent class 3 had several health problems.

The results of the present study showed that mental disorders were associated with sleep quality and QoL. Therefore, individuals in the second class (about 29%) had poor sleep quality and anxiety simultaneously. Also, members of the third class simultaneously suffered from depression, anxiety, stress, OCD, and poor sleep quality. A study in Portugal also showed the co-occurrence of sleep quality, depression, and QoL. In this study, sleep quality acted as a mediating factor between depression and QoL in the elderly concerning health and gender diversity^34^. In another study conducted in China, the prevalence of depressive symptoms in older adults was not reported as high, while sleep quality was addressed as the leading cause of depression among older adults^35^. It should be noted that there may be complex links between psychological problems with sleep quality and QoL. A study on the Chinese elderly showed that short sleep or poor sleep quality in the elderly were associated with increased anxiety in these individuals^36^.

The most significant health-related problem of the elderly in the present study was poor sleep quality with a prevalence of 50.4%. In a study conducted in nursing homes in China, the prevalence of poor sleep quality among older adults was high (67.3%) and it was also found that anxiety and poor sleep quality are correlated^37^. The prevalence of poor sleep quality among the elderly has been reported as 40% in males and 59% in females in Canada, 62.2% in India, and 39.4% in Hong Kong^38,39^. The prevalence of poor sleep quality in the present study was consistent with other studies. Factors, such as environment, pain, chronic illness, and sleep disorders, older age, symptoms of depression and anxiety, and poor social support could cause poor sleep quality in the elderly. Finally, poor sleep quality can increase the risk of heart disease, depression, falls, and accidents among older adults^37,40^.

The present study showed that the female gender significantly increased the odds of being in the second and third classes compared to the first class. Thus, older women were more likely than older men to have poor QoL, sleep quality, and mental disorders. Studies conducted on the elderly indicate that the female gender was linked with many health problems, such as poor sleep quality and QoL^8,41^. In addition, a study on the Chinese elderly found that women were more likely to report sleep disorders than men, due to doing household chores, which put them at greater risk for problems such as sleep disorders^42^. In another study, poor sleep quality was more prevalent in the female elderly^43^.

This study showed that living in urban significantly increases the odds of membership in the second and third classes compared to the first class. A study showed that elderly people living in rural areas had more sleep disorders than urban residents due to low economic situation and heavy work in the field and insufficient access to sleep clinics and sleep training in rural areas^42^. In a review, depression was higher among urban elderly than rural elderly^44^. Another study found that living in the urban area, female gender, aging, and illiteracy increased the risk of depression in the elderly^45^. Given that the probability of mental disorders was higher in the second and third classes, so it can be said that living in the city was significantly more associated with a higher prevalence of mental disorders. Further studies in this regard can help clarify these relationships.

The present study also showed that illiteracy increased the odds of class 3 membership compared to class 1. Lou et al.^46^ found that older people with lower education had poorer sleep quality than those with higher education. Also, Li et al.^43^ showed that older adults with low education had poor sleep quality. Given that education causally affects the health of older males and their cognitive skills^47^, as well as the strong link between education and the general health of the population^48^, it seems that education-related policies can be considered as indirect health policies. Therefore, among older adults, the necessary knowledge and awareness of these people about health can be improved through establishing educational workshops and interventions.

In the present study, there were some limitations, including the inability of this study to be generalized to all the elderly population of the province or country, due to implementation in a city and also the self-report of the collected information that may not be reliable in some cases.

## Conclusion

In this study, latent classes of health-related problems among the elderly were identified and the effect of demographic variables on the membership of individuals in different classes was investigated. In general, the results of our study showed that the co-occurrence of health problems in 33.2% of the elderly was observed in various forms. Given the important role of anxiety and low sleep quality in the classification of the elderly, effective interventions are necessarily required to reduce anxiety and improve sleep quality among older adults. The results of this study can be used in prioritizing health programs for the elderly and emphasizing high-risk groups.

## Data Availability

The datasets generated during the current study are not publicly available since they will contain patient data and the informed consent agreement does not include sharing data publicly. However, the data are available from the corresponding author on reasonable request.
